# The effect of intravenous glucose solutions on neonatal blood glucose levels after cesarean delivery

**DOI:** 10.1007/s00540-012-1516-1

**Published:** 2012-11-11

**Authors:** Isao Fukuda, Hideo Matsuda, Shinya Sugahara, Tomiei Kazama

**Affiliations:** 1 Department of Anesthesiology, National Hospital Organization Tokyo National Hospital, 3-1-1 Takeoka, Kiyose, Tokyo 204-8585 Japan; 2 Department of Obstetrics and Gynecology, Matsuda Perinatal Clinic, Saitama, Japan; 3 Department of Anesthesiology, National Defense Medical College, Saitama, Japan

**Keywords:** 1 % glucose acetated Ringer’s solution, Blood glucose, Perioperative infusion

## Abstract

**Purpose:**

Intravenous solutions are often administered to the mother on the day of a cesarean delivery to minimize the effect of preoperative fasting or to stabilize the hemodynamics. Different intravenous solutions contain varying amounts of glucose, and rapid administration may lead to hypoglycemia in the neonate. We conducted a study to compare blood glucose levels of the mother and the fetus/neonate after they were rapidly given a Ringer’s solution containing 0, 1, or 5 % glucose. The effect of the glucose load that these intravenous solutions impose during cesarean delivery has not been fully reported. Therefore, we compared the effect of 0 % (Group I, *n* = 15), 1 % (Group II, *n* = 15), and 5 % (Group III, *n* = 15) glucose acetated Ringer’s solutions on maternal and umbilical blood glucose levels to determine the optimal glucose concentration.

**Methods:**

Once the patients were in the operating room, the intravenous solutions were administered before delivery. The primary endpoint was changes in umbilical blood glucose levels and minimum neonatal blood glucose levels, and the secondary endpoint was the proportion of neonates who received a glucose infusion.

**Results:**

Maternal blood glucose levels before and after intravenous infusion were 79.2 ± 12.2 and 74.6 ± 4.6 in Group I, 81.2 ± 12.9 and 103.3 ± 11.2 in Group II (*P* < 0.001), and 82.3 ± 8.7 and 252.5 ± 41.8 in Group III (*P* < 0.001). Umbilical blood glucose levels were 53.9 ± 10.2 in Group I, 80.8 ± 13.7 in Group II, and 181.8 ± 22.2 in Group III (*P* < 0.01: Group I vs. Group II and *P* < 0.01: Group II vs. Group III) (*P* < 0.001: Group I vs. Group III). Minimum neonatal blood glucose levels measured up to 8 h after birth were 35.7 ± 9.6 in Group I, 49.8 ± 10.8 in Group II, and 29.2 ± 7.5 in Group III. Neonatal hypoglycemia requiring glucose before the first milk feeding occurred in 6 neonates whose mothers were in Group I, 3 in Group II, and 9 in Group III, indicating a trend towards less neonatal hypoglycemia in Group II.

**Conclusions:**

The use of 1 % glucose acetated Ringer’s solution did not induce hyperglycemia in the mother and it was able to maintain appropriate blood glucose levels in the fetus.

## Introduction

Intravenous glucose solutions may be given to patients who undergo cesarean deliveries to provide the mother and fetus with energy, and glucose-free crystalloid solutions are given to maintain hemodynamics. However, glucose load from preoperative intravenous solutions may result in hyperglycemia or hypoglycemia in the fetus.

Anesthesia induction often causes an abrupt change in hemodynamics, and such change, especially a decrease in blood pressure, harms both mother and fetus. To reverse the change, a vasopressor is promptly administered, and rapid intravenous infusion is often performed to have a faster effect. However, depending upon the intravenous solutions used, rapid administration may result in maternal and fetal hyperglycemia, which eventually could lead to reflex hypoglycemia in the neonate [1]. The optimal glucose concentration for intravenous solutions that would be effective for maintaining a safe range of blood glucose both in mothers and fetuses has not been established. Normally, mothers with blood glucose levels of 40 mg/dL or lower and fetuses with levels of 30 mg/dL or lower are diagnosed as having hypoglycemia [2]. A neurophysiological study indicates that developmental disorders will occur if the neonate’s blood glucose level becomes 2.6 mmol/L (equivalent to 48 mg/dL) or lower, which is consistent with the diagnostic criterion previously mentioned [3]. Intravenous solutions of acetated Ringer’s used during surgery can be generally categorized based on glucose load into the following 3 types: 0 % acetated Ringer’s solution (no glucose, 500 mL), 1 % acetated Ringer’s solution (5 g glucose, 500 mL), and 5 % acetated Ringer’s solution (25 g glucose, 500 mL) (Table 1). The effect of the glucose load that these intravenous solutions impose during cesarean delivery has not been fully reported. Therefore, we compared the effect of 0, 1, and 5 % glucose acetated Ringer’s solutions on maternal and umbilical blood glucose levels to determine the optimal glucose concentration.

**Table 1:** Composition of test solutions

Name	Group name	Na^+^	K^+^	Ca^+^	Mg^+^	Cl^−^	Gluconate^−^	Citrate^3−^	Acetate^−^	Glucose (%)	Osmotic pressure ratio
		(mEq/L)									
0 % Glucose acetated Ringer’s solution	Group I	130	4	3	–	109	–	–	28	–	Approx. 1
1 % Glucose acetated Ringer’s solution	Group II	140	4	3	2	115	3	6	25	1	Approx. 1
5 % Glucose acetated Ringer’s solution	Group III	130	4	3	–	109	–	–	28	5	Approx. 2

## Patients and methods

The study was approved by the Institutional Ethics Committee of the National Defense Medical College. The patients were fully informed about the study, and written informed consent was obtained. Forty-five pregnant women scheduled for elective cesarean delivery were included. Patients with gestational diabetes or pregestational diabetes or those receiving intravenous solutions with glucose due to preoperative complications, such as threatened premature labor, were excluded.

**Fig. 1:**
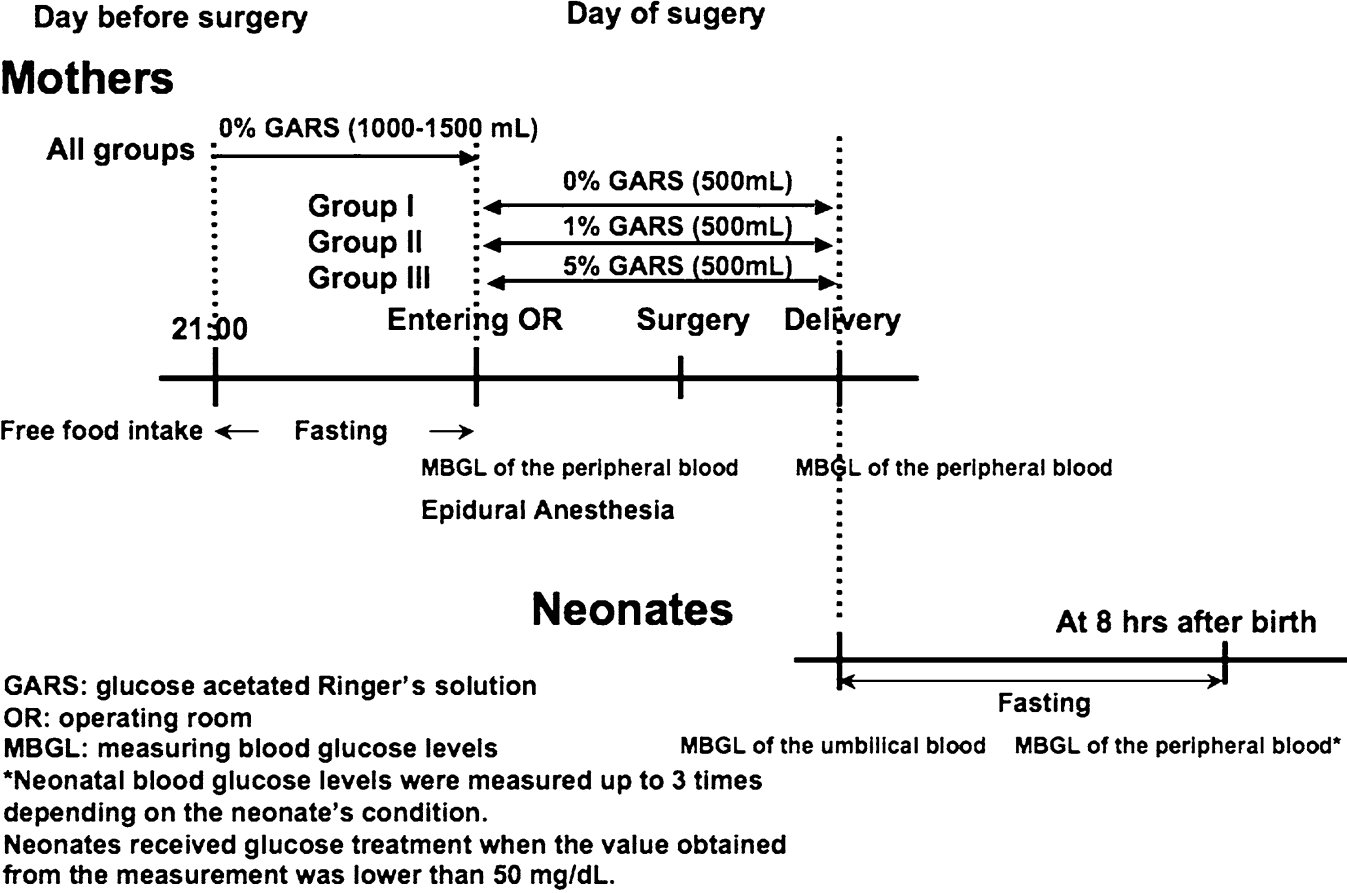
Time schedule of the study

The 45 patients were randomly assigned to 3 groups: 0 % glucose acetated Ringer’s solution (Group I), 1 % glucose acetated Ringer’s solution (Group II), and 5 % glucose acetated Ringer’s solution (Group III). All patients received an infusion of 0 % glucose acetated Ringer’s solution (1000–1500 mL) via the forearm the day before their surgery was scheduled; preoperative fasting started at 21:00 the night before the scheduled cesarean delivery section. Patients were not given any other medications, including anesthetics, before their scheduled delivery (Fig. 1).

Lumbar epidural anesthesia was performed by injecting 15 mL of 1 % ropivacaine via the L3-4 interspace. A Teflon needle was placed in the radial artery to monitor arterial pressure and to take maternal blood samples. Each group received 500 mL of the test solution, and administration was completed before delivery. As for glucose load; Group I received 0 g glucose; Group II, 5 g glucose; and Group III, 25 g. To determine glucose levels, maternal blood samples were obtained before intravenous infusion and immediately after delivery; umbilical blood was obtained immediately after delivery to measure glucose levels and pH. The neonate’s body weight was also measured and the Apgar score determined by a neonatologist. Neonates with a minimum blood glucose level of 50 mg/dL or lower within 8 h of delivery were given a glucose infusion.

Results were expressed as mean ± SD and median (minimum–maximum). One-way analysis of variance (two-sided at *α* = 0.05) and Chi-squared test were performed for statistical analyses by using Prism 5 for Mac OS X (1992–2008, GraphPad Software, Inc.). If one-way ANOVA gave a *P* value less than 0.05, a post hoc test (Dunnett’ method) was performed. Differences in the patient’s demographic characteristics were considered significant at the 0.15 level.

## Results

Cesarean deliveries were performed in patients with breech presentation (22.2 %), repeated cesarean section (CS) (40.0 %), maternal request (0.04 %), and after myomectory CS (33.3 %).

For the mothers, there were statistically significant differences in age, gestational week, and anesthesia time; whereas there were no differences in height, body weight, or amount of bleeding or oxytocin received (Table 2). For the neonates, there were no differences in body weight or Apgar scores (Table 3). Before and after the start of the intravenous infusion, the maternal blood glucose levels (mg/dL) were 79.2 ± 12.2 and 74.6 ± 4.6 in Group I, 81.2 ± 12.9 and 103.3 ± 11.2 in Group II, and 82.3 ± 8.7 and 252.5 ± 41.8 in Group III (Table 4). Group II and Group III showed significant differences in blood glucose levels before and after the start of the intravenous infusion (Group II, *P* < 0.001; Group III, *P* < 0.001), and between-group differences were noted; significant differences between the 3 groups were also found (Group I vs. Group II, *P* < 0.001; Group II vs. Group III, *P* < 0.001; Group I vs. Group III, *P* < 0.001; one-way ANOVA, *P* < 0.05). Immediately after delivery, umbilical blood glucose levels (mg/dL) were 53.9 ± 10.2 in Group I, 80.8 ± 13.7 in Group II, and 181.8 ± 22.2 in Group III (Table 5). Of the 3 groups, Group III had the highest blood glucose levels, and there were significant differences between the 3 groups (Group I vs. Group II, *P* < 0.01; Group II vs. Group III, *P* < 0.01; Group I vs. Group III, *P* < 0.001; one-way ANOVA, *P* < 0.05). The minimum neonatal blood glucose levels within 8 h after birth (mg/dL) were 35.7 ± 9.6 in Group I, 49.8 ± 10.8 in Group II, and 29.2 ± 7.5 in Group III (Table 5). Group I and Group III showed significantly lower minimum blood glucose levels within 8 h after birth than in Group II (Group I vs. Group II, *P* < 0.05; Group II vs. Group III, *P* < 0.01).

**Table 2:** Baseline maternal characteristics

	0 % Glucose acetated Ringer’s solution Group I (*n* = 15)	1 % Glucose acetated Ringer’s solution Group II (*n* = 15)	5 % Glucose acetated Ringer’s solution Group III (*n* = 15)	*P*
Age (year)	34.5 ± 2.6	33.5 ± 3.6	31.9 ± 4.1	0.14
Height (cm)	158.4 ± 4.2	158.0 ± 4.9	157.0 ± 6.3	0.75
Body weight (kg)	62.6 ± 6.6	61.7 ± 6.5	61.1 ± 6.0	0.79
Gestational week	36.9 ± 1.7	37.4 ± 2.7	36.1 ± 1.7	0.13
Amount of oxytocin used (U)	23.3 ± 6.7	22.7 ± 14.5	21.0 ± 6.7	0.80
Blood loss (g)	1110 ± 457.4	912.7 ± 354.7	895.7 ± 378.9	0.84
Anesthetic time (min)	117.6 ± 35.2	115.3 ± 41.6	111.1 ± 40.8	0.07

Mean ± SD

One-way ANOVA, *α* = 0.15

**Table 3:** Baseline neonatal characteristics

	0 % Glucose acetated Ringer’s solution Group I (*n* = 15)	1 % Glucose acetated Ringer’s solution Group II (*n* = 15)	5 % Glucose acetated Ringer’s solution Group III (*n* = 15)	*P*
Neonatal body weight (g)^a^	2668.0 ± 460.3	2685 ± 593.1	2570.0 ± 566.6	0.53
Apgar score 1 min^b^	9 (8–9)	9 (7–9)	9 (8–9)	0.53
Apgar score 5 min^b^	10 (9–10)	10 (9–10)	10 (9–10)	0.72
Fetal pH^b^	7.428 (7.389–7.466)	7.391 (7.310–7.452)	7.386 (7.327–7.431)	0.36

One-way ANOVA, α = 0.15

^a^Mean ± SD

^b^Mean (minimun–maximum)

**Table 4:**
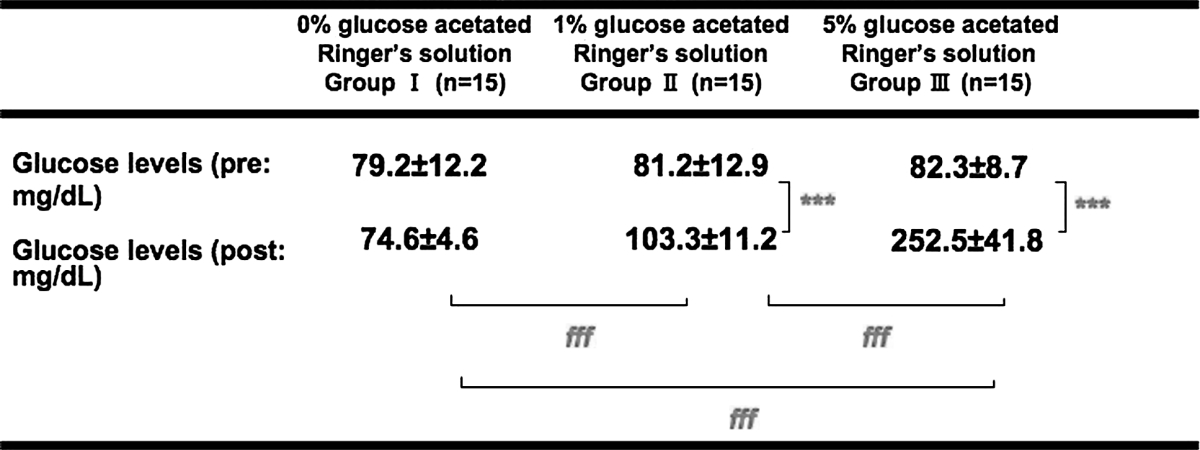
Blood glucose levels of mothers

One-way ANOVA, α = 0.05

Mean ± SD

*** *P* < 0.001 for pre-infusion versus post-infusion

fff *P* < 0.001 for each groups

**Table 5:**
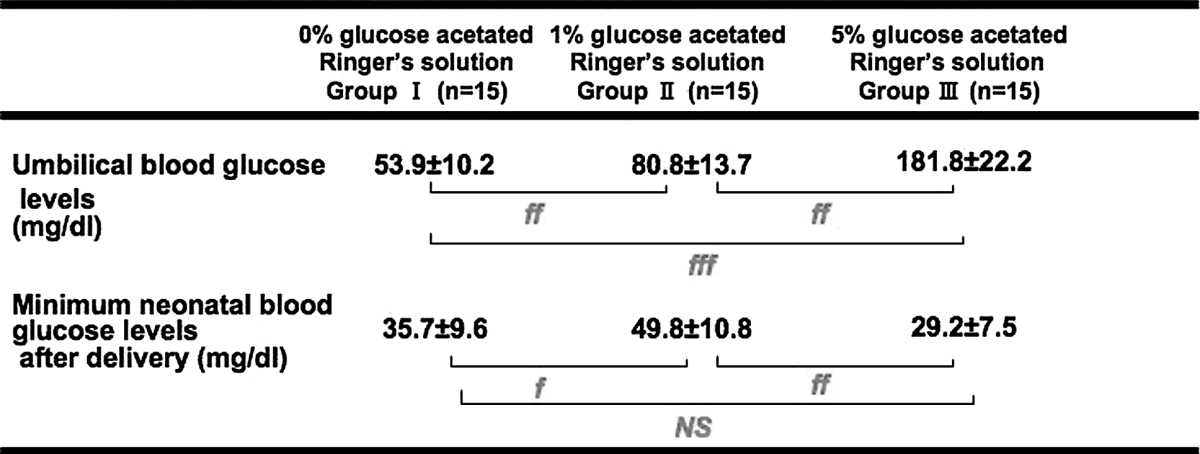
Blood glucose levels of fetuses and neonates

One-way ANOVA, α = 0.05

Mean ± SD

*NS* not significant

f *P* < 0.05 for each groups

ff *P* < 0.01for each groups

fff *P* < 0.001 for each groups

Based on the reference range of our institution, a neonate with a minimum blood glucose level of 50 mg/dL (2.8 mmol/L) or lower measured within 8 h after birth was diagnosed as having neonatal hypoglycemia and given treatment with a glucose infusion. In this study, 6 neonates in Group I, 3 in Group II, and 9 in Group III received a glucose infusion before the first milk feeding; neonates in Group II showed a trend towards a less frequent glucose infusion (*P* = 0.082) (Table 6).

**Table 6:** Number of neonates who received treatment

	0 % Glucose acetated Ringer’s solution Group I (*n* = 15)	1 % Glucose acetated Ringer’s solution Group II (*n* = 15)	5 % Glucose acetated Ringer’s solution Group III (*n* = 15)	*P*
No. of neonates who received glucose infusion therapy after delivery	6	3	9	0.082

Chi-squared test, α = 0.05

## Discussion

In our study, we compared maternal, umbilical, and neonatal (8 h after birth) blood glucose levels to investigate the optimal glucose infusion in neonates. Baseline maternal characteristics showed statistically significant differences in age, gestational week, and anesthesia time in 3 groups; however, they were not considered clinically important. As a result, those who received 1 % glucose (Group II) had significantly lower glucose levels than other groups. There were significant differences in umbilical blood glucose and minimum neonatal blood glucose levels within 8 h after birth between groups receiving Ringer’s solutions with different glucose concentrations (0, 1, and 5 %).

Generally, cesarean deliveries are performed under local anesthesia such as epidural or spinal anesthesia. However, local anesthesia often results in a decrease in blood pressure because of sympathetic nervous blockade, which should be promptly reversed because such hypotension will harm not only the mother but also the fetus. To stabilize the blood pressure, a vasopressor is generally administered, and rapid infusion of an intravenous solution is often concomitantly used to have a faster effect. Currently, several solutions with different glucose concentrations are available for cesarean delivery, but are characterized by no uniformity.

Glucose passes between the mother and fetus through the placenta by diffusion, but its correlation coefficient is high, and there is no limit to the amount of glucose that can be passed [4]. Normally, the difference in blood glucose levels between the mother and fetus is within 20 mg/dL, but if the mother receives a rapid glucose-containing infusion, the umbilical glucose levels have been reported to increase to 300 mg/dL or more [5].

The use of glucose infusion in cesarean delivery has not been established.

Carbohydrate metabolism plays a central role in fetal metabolism. In 1959, Dawes and others [5] described the positive correlation between the period that a fetus can survive with anoxia and the amount of glycogen that accumulates in the heart [6]. During delivery, energy is derived from glucose via the Emden–Meyerhof pathway. When the mother–fetal condition is normal, increased fetal blood glucose is not as problematic as in instances of fetal distress, when providing glucose has been reported to benefit the fetus [7, 8]. If the fetus is stressed, catecholamines secreted from the adrenal gland and nerve endings enhance key enzymes of the Emden–Meyerhof pathway, degrade glycogen, and increase the blood glucose level. Furthermore, glucose load from intravenous solutions results in fetal hyperglycemia, and the excess glucose is not metabolized in the Krebs cycle, which may increase blood lactic acid and ultimately lead to lactic acidosis [1]. Especially rapid and large-dose administration should be contraindicated in cesarean delivery because it can result in maternal and fetal hyperglycemia, which may eventually lead to reflex hypoglycemia in the neonate [9–11].

In Japan, hypoglycemia in infants is generally defined as a blood glucose level of 30 mg/dL (1.7 mmol/L) or lower. However, as mentioned above, at our institution, for safety, the definition of hypoglycemia in neonates is a minimum blood glucose level of 50 mg/dL (2.8 mmol/L) or lower within 24 h after delivery [2]. Deshpande and colleagues [3] have reported that a blood glucose level of 48 mg/dL (2.6 mmol/L) or lower in neonates may lead to developmental disorders. Another study recommends that neonates with any abnormal clinical signs should be treated to have a blood glucose level of 45 mg/dL (2.5 mmol/L) or higher [12].

As for blood glucose levels of the neonate after birth, Marom and colleagues showed that neonates had a level of 70.9 ± 9.7 mg/dL when preoperative fasting lasted at least 6 h for mothers scheduled to have a cesarean delivery [13]. On the other hand, hypoglycemia may occur because pregnant women who are scheduled for elective cesarean delivery are required to fast after 21:00 the night before surgery. If the neonate’s minimum blood glucose level is 50 mg/dL or lower within 8 h of birth, parenteral nutrition with a glucose-containing solution is begun to ensure the neonate’s safety. In our study, neonates in Groups I and III were more likely to receive therapy than neonates in Group II. As a result, blood glucose levels in neonates in those 2 groups were significantly higher (Table 3). Group I mothers had hypoglycemia (no glucose load), which led to hypoglycemia in the neonate. In contrast, neonates in Group III had hyperglycemia immediately after birth. The glucose load in Group III mothers led to hyperglycemia and, subsequently, hyperinsulinemia in the fetus. In terminal gestation, mean immunoreactive plasma insulin (IRI) is 5 μU/mL, which increases along with the elevation in fetal blood glucose levels due to maternal glucose load and fetal stress: there is a positive correlation between fetal blood glucose and fetal plasma insulin [14–16]. When the umbilical cord is clamped at delivery, however, glucose supply is terminated abruptly, leading to severe hypoglycemia due to hyperinsulinemia.

As a limitation of our study, we did not measure maternal, fetal, or neonatal insulin levels directly, which needs to be further investigated in future studies. In our hospital, glucose infusion is given as a prevention of neonatal hypoglycemia, but clinically speaking, first milk feeding may be started earlier. Also, this study was conducted as an exploratory study, and the sample size was not calculated, which might have led to the inconclusive results regarding glucose infusion in neonates with no statistical difference due to lack of statistical power. Further clinical studies with appropriate sample sizes may be conducted in the future.

The true purpose of our study is to further investigate how patients with gestational diabetes or pregestational diabetes respond to intravenous glucose solution. As a next step, we are planning to conduct a study with patients with pregestational diabetes.

In conclusion, our study results suggest that administration of 1 % glucose acetated Ringer’s solution (500 mL) during cesarean delivery is a reasonable treatment to maintain appropriate glucose levels in neonates.
